# Supplementary material for: Dihydromyricetin Enhances Exercise-Induced GLP-1 Elevation through Stimulating cAMP and Inhibiting DPP-4
Source: Nutrients. 2022 Nov 1;14(21):4583. doi: 10.3390/nu14214583 (PMC9656859; doi:10.3390/nu14214583)
Supplement: Supplementary file 1 [file nutrients-14-04583-s001.zip › supplement material.pdf]

## Supplementary Figures

## Figure legends

Fig. S1 Effects of two-week swimming training on body weight and organ weights. (A) Initial body weight and final body weight. (B) Body weight gain= final body weight-initial body weight. (C) 24 food intakes. (D) 24 water intakes. (E) Gastrocnemius muscle mass/body weight. (F) Tibialis anterior muscle mass/body weight. (G) Soleus muscle mass/body weight. (H) iWAT weight/body weight. Data are expressed as the mean ± SEM. *P < .05, **P < .01 vs CON.

Fig. S2 Swimming training alters gut microbiota. (A, B) Relative abundance and Wilcoxon rank-sum test bar plot on Phylum (A) and Genus (B) level between EXE_w2 and CON_w2 groups. (C) Linear discriminant analysis (LDA) scores derived from the LEfSe analysis, showing the biomarker taxa (LDA score of >2 and a significance of P < .05, as determined by the non-parametric factorial Kruskal-Wallis (KW) sum-rank test). (D) Community heatmap showing the abundance of the top 50 genus altered by swimming training. Data are expressed as the mean ± SEM. *P < .05, **P < .01, and ***P < .001.

Fig. S3 Two-week swimming training alters short-chain fatty acid profiles. Pearson correlation analysis of SCFAs with gut microbiota on genus level. Red indicates a positive correlation, blue indicates a negative correlation, and white indicates no correlation. *P < .05, and **P < .01.

## Supplemental Experimental Procedures

### Western blot analysis

Protein samples were extracted from colon samples. Colon samples were homogenized in T-PER Tissue Protein Extraction Reagent (Invitrogen) containing one protease inhibitor cocktail tablet (Roche Applied Science, Germany) and one PhosSTOP phosphatase inhibitor cocktail tablet (Roche Applied Science, Germany) per 10 mL of protein extraction reagent. The protein concentration was determined by the bicinchoninic acid (BCA) method with an Enhanced BCA Protein Assay Kit

(Beyotime, China). Briefly, equal quantities of protein were electrophoretically separated through 12% SDS-PAGE, then electro-transferred to PVDF membranes using a wet transfer method, blocked for 1h using 5% BSA at room temperature, incubated with primary antibodies: mouse Anti-GLP-1 monoclonal antibody (1:1000, Santa Cruz Biotechnology), overnight at 4 °C and relative secondary antibodies for 1h at room temperature. β-actin protein was used as a loading control for further analysis. The chemiluminescence of target protein was detected using Immobilon Western Chemiluminescent HRP Substrate (Millipore). Gel images were acquired using fusion fx5 molecular imager (Vilber Lourmat) and gray values were calculated by Image J software.

**Data processing method of 16S rRNA sequencing**

Using UPARSE version 7.1, the raw 16S rRNA gene sequencing reads were demultiplexed, quality-filtered merged and clustered into OTUs with a 97% similarity cutoff, and the chimeric sequences were then identified and eliminated. The taxonomy of each OTU representative sequence was analyzed by RDP Classifier version 2.2 against the SILVA database (version138) with a confidence threshold of 0.7. Data processing was carried out on the online platform of Majorbio Cloud Platform (www.majorbio.com).

**Assessment of GLP-1 secretion from GLUTag cells**

GLP-1 secretion was assessed by ELISA from GLUTag cells, which had proven to be a useful model for the stimulation of GLP-1 secretion. Cells were seeded with culture medium into 24-well culture plates, two days previous to the experiment. On the day of the experiment, cells were washed twice with 500 μL secretion medium, then grown in secretion medium to starve the cells for 1 h. Secretion medium was a glucose-free KRBB (138 mM NaCl, 5.6 mM KCl, 2.6 mM MgCl2, 4.2 mM NaHCO3, 1.2 mM NaH2PO4 and 10 mM hepes) contained with 0.5% (w/v) BSA. Experiments were carried out by incubating the cells with or without DHM (Sigma-Aldrich) in 500 μL of secretion medium for 120 minutes at 37°C and 5% CO2. DHM was initially

dissolved in dimethyl sulfoxide (DMSO) to prepare a stock solution (100 mM). At the end of the incubation time, the medium and cell lysate were collected, frozen at -80°C for further analysis.
