# Supplementary figures and images for: Dihydromyricetin Enhances Exercise-Induced GLP-1 Elevation through Stimulating cAMP and Inhibiting DPP-4
Source: Nutrients. 2022 Nov 1;14(21):4583. doi: 10.3390/nu14214583 (PMC9656859; doi:10.3390/nu14214583)

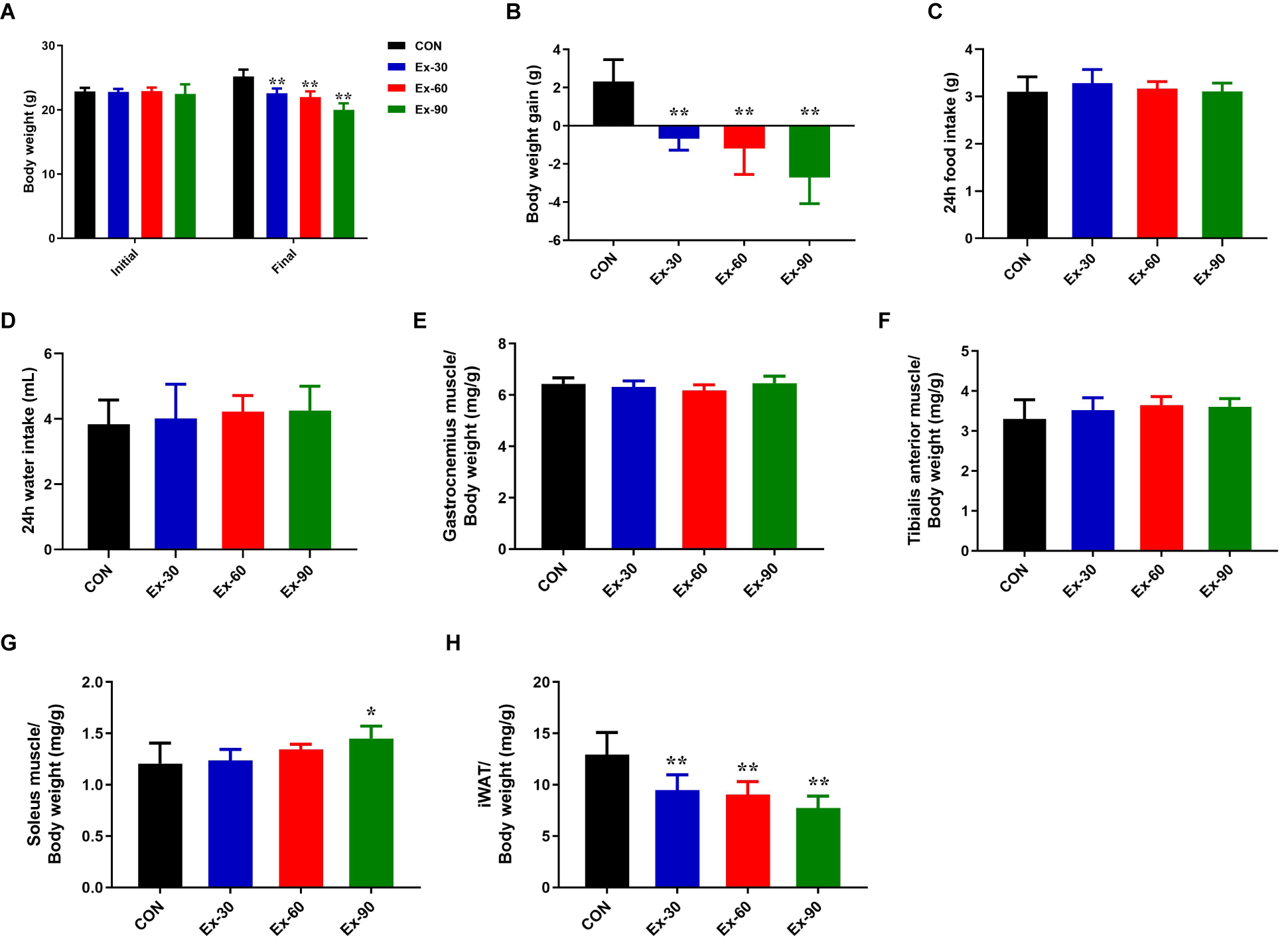

Supplement: Supplementary file 1 [file nutrients-14-04583-s001.zip › figure S1.tif]

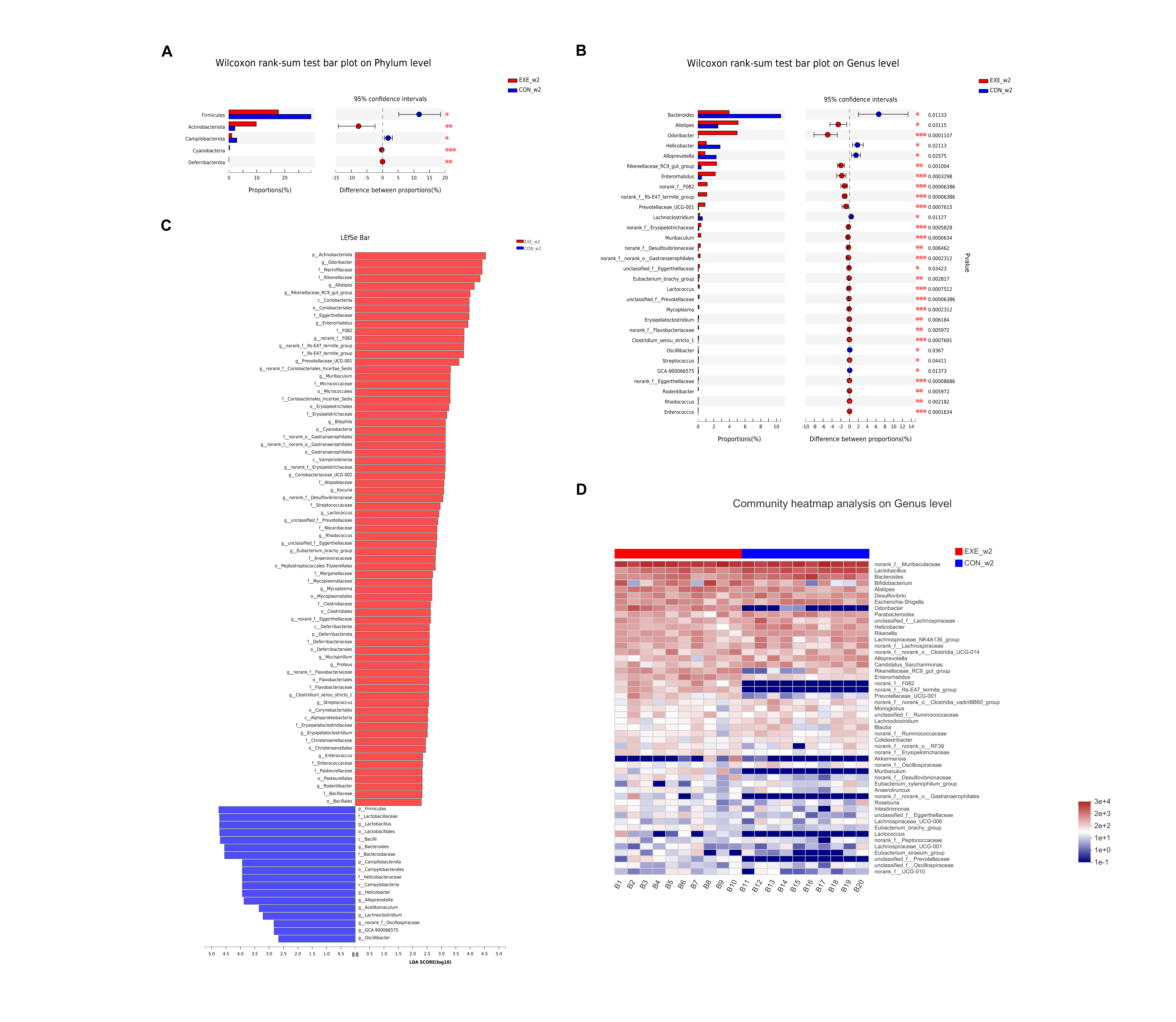

Supplement: Supplementary file 1 [file nutrients-14-04583-s001.zip › Figure S2.tif]

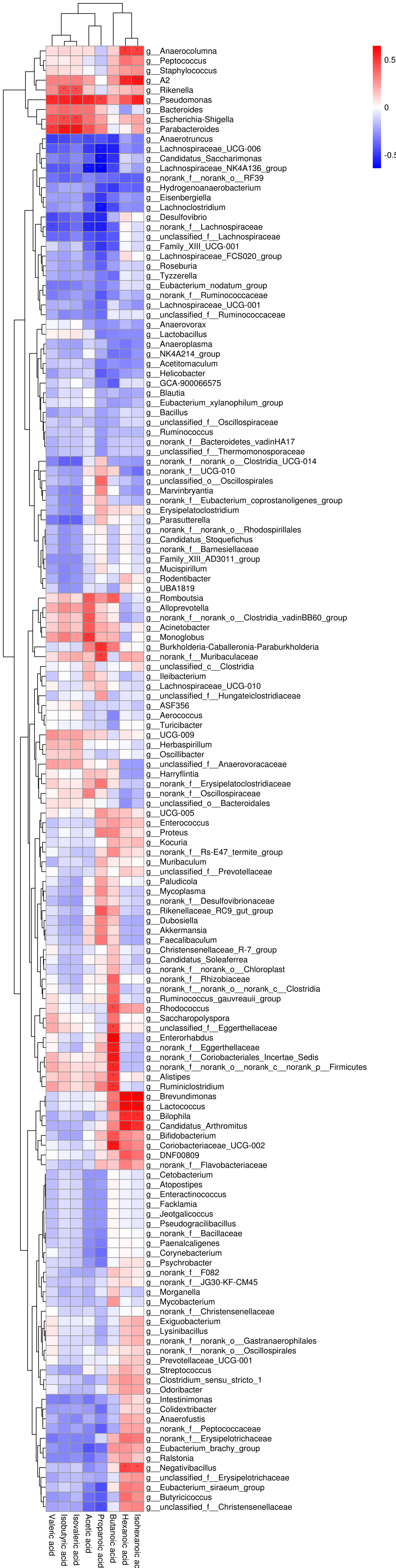

Supplement: Supplementary file 1 [file nutrients-14-04583-s001.zip › Figure S3.pdf]
